# The benefit of adding polygenic risk scores, lifestyle factors, and breast density to family history and genetic status for breast cancer risk and surveillance classification of unaffected women from germline *CHEK2* c.1100delC families

**DOI:** 10.1016/j.breast.2024.103724

**Published:** 2024-04-12

**Authors:** Maartje A.C. Schreurs, Teresa Ramón y Cajal, Muriel A. Adank, J. Margriet Collée, Antoinette Hollestelle, Jeroen van Rooij, Marjanka K. Schmidt, Maartje J. Hooning

**Affiliations:** a Department of Medical Oncology, Erasmus MC Cancer Institute, Rotterdam, the Netherlands; b Familial Cancer Clinic, Medical Oncology Service, Hospital Sant Pau, Barcelona, Spain; c Department of Clinical Genetics, Netherlands Cancer Institute - Antoni van Leeuwenhoek Hospital, Amsterdam, the Netherlands; d Department of Clinical Genetics, Erasmus MC Cancer Institute, Rotterdam, the Netherlands; e Department of Internal Medicine, Erasmus MC, Rotterdam, the Netherlands; f Division of Molecular Pathology, Netherlands Cancer Institute – Antoni van Leeuwenhoek Hospital, Amsterdam, the Netherlands

**Keywords:** *CHEK2* c.1100delC, Breast cancer, Risk prediction

## Abstract

To determine the changes in surveillance category by adding a polygenic risk score based on 311 breast cancer (BC)-associated variants (PRS_311_), questionnaire-based risk factors and breast density on personalized BC risk in unaffected women from Dutch *CHEK2* c.1100delC families.

In total, 117 unaffected women (58 heterozygotes and 59 non-carriers) from *CHEK2* families were included. Blood-derived DNA samples were genotyped with the GSAMDv3-array to determine PRS_311_. Lifetime BC risk was calculated in CanRisk, which uses data from the Breast and Ovarian Analysis of Disease Incidence and Carrier Estimation Algorithm (BOADICEA). Women, were categorized into three surveillance groups.

The surveillance advice was reclassified in 20 (34.5%) heterozygotes and 21 (35.6%) non-carriers after adding PRS_311_. Including questionnaire-based risk factors resulted in an additional change in 11 (20.0%) heterozygotes and 8 (15.1%) non-carriers; and a sub-analysis showed that adding breast density on top shifted another 9 (23.1%) heterozygotes and 5 (27.8%) non-carriers. Overall, the majority of heterozygotes were reclassified to a less intensive surveillance, while non-carriers would require intensified surveillance.

The addition of PRS_311_, questionnaire-based risk factors and breast density to family history resulted in a more personalized BC surveillance advice in *CHEK2*-families, which may lead to more efficient use of surveillance.

## Highlights

• Lifetime breast cancer risk in unaffected *CHEK2* heterozygotes and familial non-carriers was calculated using CanRisk.
• Risks ranged from 22.1 to 51.7% in heterozygotes and 10.7–31.0% in non-carriers based on family history alone.
• Adding PRS_311_ caused the largest shift in risk prediction, followed by breast density and questionnaire-based risk factors.
• Risk stratifications were similar among distant relatives, not supporting modified cascade screening in *CHEK2* families.

## 1 Introduction

The pathogenic germline *CHEK2* c.1100delC variant is the most prevalent breast cancer (BC) predisposition variant in the Netherlands. It is present in about 1% of the general Dutch population, increasing to 2.5% in unselected BC cases and up to 5% in familial BC cases [1,2]. Depending on family history (FH), the *CHEK2* variant confers a lifetime BC risk (LTBCR, defined as the risk of developing BC from age 20 to 80) of 20–55% [[1], [2], [3], [4], [5], [6], [7]], compared to the population-based BC risk of 14% [8]. As a result, relatives of the index case are eligible for genetic counselling. However, over time, discussions have been ongoing to what extent relatives should be eligible for genetic counselling in families with moderate-risk pathogenic variants. Currently, in the Netherlands, only female first and second degree relatives are eligible for *CHEK2* testing (modified cascade screening) [9]. Based on personal genetic status and FH, an age-specific breast surveillance program is advised [10].

However, there are other known BC risk modifiers, which are mostly currently not yet taken into account during counselling. A more personalized BC risk estimation and surveillance advice would be preferable, especially for young unaffected women. Several comprehensive BC risk prediction models have been developed over the last decade, including the Breast and Ovarian analysis of Disease Incidence and Carrier Estimation Algorithm (BOADICEA) [11], which has been incorporated into the CanRisk prediction tool [12,13]. This model has recently been validated in Dutch women from the general population [14], and includes information on FH [15], genetic testing results, the polygenic risk score (PRS) [16], BMI [17], alcohol consumption [18,19], hormonal factors [20,21], and breast density (BD) [15].

While evidence on the clinical utility of incorporating the PRS in risk prediction of *CHEK2* carriers is still lacking, several studies have shown the added improvement of risk stratification [11,16,[22], [23], [24], [25]]. Firstly, risk effects conferred by *CHEK2* and 77 common variants acted multiplicatively [16] and more recently, two studies including variant status, individual clinical variables and PRS showed meaningful shifts in LTBCR in *CHEK2* carriers regardless of a family history of BC [24,25]. In the familial cancer setting, one study on BC risk prediction of non-*BRCA1/2* Dutch families showed that the addition of PRS_313_ changed clinical management in 58% of the *CHEK2* carriers [26]. However, a recent study found linkage between two SNPs located on the 22nd chromosome (chr22:29203724:C/T and chr22:29551872:A/G) and the *CHEK2* c.1100delC variant, and therefore using PRS_313_ would result in an overestimation of BC risk in heterozygotes. It is advised to exclude these two SNPs from the PRS and use the PRS_311_ for CHEK2 families [27].

Within this study, we aimed to investigate the benefit of incorporating PRS_311_, BD and questionnaire-based risk factors (QRFs: including anthropometric information, lifestyle factors, and hormonal factors) in the CanRisk tool one-by-one by evaluating the effect on individual BC risk prediction and changes in screening surveillance advice for unaffected women from *CHEK2* families. Adding this next to FH and *CHEK2* status is expected to result in a more accurate and personalized risk prediction and consequently, more appropriate surveillance advice. We evaluated the impact of using the PRS_311_ versus the PRS_313_ next to FH and *CHEK2* status on BC risk stratification. In addition, we compared risk stratification for first-, second-, and third-degree relatives, contributing to the ongoing discussion on cascade screening for these families.

## 2 Materials and methods

### 2.1 Study cohort

We used retrospective cohort data from women participating in the nation-wide Hereditary Breast and Ovarian cancer study Netherlands (Hebon) study. The selected women were counselled at Erasmus MC or AVL-NKI hospital for the *CHEK2* c.1100delC variant. All women had signed informed consent. The Hebon study has been approved by the Medical Ethics Committee of the Erasmus MC and the Institutional Review Board (IRB) of the AVL-NKI.

**Fig. 1:**
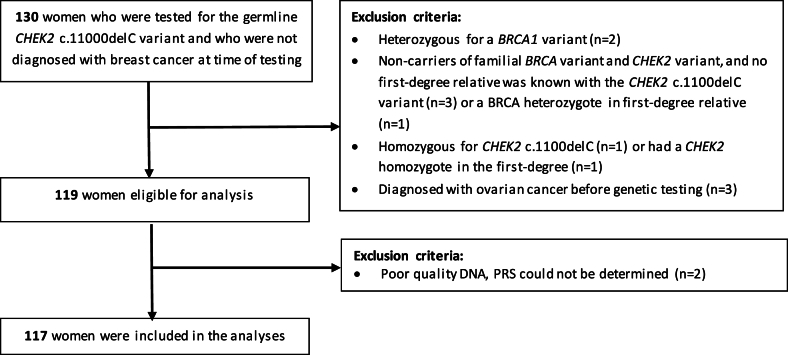
flowchart of women excluded from this study.

We selected 130 women from 93 *CHEK2* families who were unaffected at the time of testing and of whom a DNA sample was available for PRS testing. After excluding six women where a g*BRCA1* PV was found in themselves or their family, two *CHEK2* homozygotes and three women diagnosed with ovarian cancer before testing, 119 women were eligible for this study. After genotyping, two women were excluded as the PRS could not be determined, resulting in a total of 117 women (58 heterozygotes and 59 non-carriers) included in the analyses (Fig. 1).

### 2.2 Data collection

#### 2.2.1 Questionnaire-based risk factors

Women participating in the Hebon study were asked to complete a detailed questionnaire containing information on risk factors, such as lifestyle factors (alcohol consumption, smoking, physical activity, BMI), hormonal factors (age at menarche and menopause, parity, exogenous hormone use), medical information (e.g. cancer diagnosis, treatment) and preventive strategies (e.g. prophylactic mastectomy). In this study, we will refer to BMI, alcohol consumption and hormonal factors as QRFs. For ten women (four heterozygotes, six non-carriers), no information on any of the addressed QRFs was available.

#### 2.2.2 Pedigrees and medical information

Pedigrees were collected from all participating women. For the LTBCR calculations, defined as the risk of developing BC from age 20 to 80, FH included all known first-degree to third-degree relatives of genotyped individuals. In addition, missing information from the questionnaire, breast surveillance recommendations given after genetic testing, and BD information (ACR BI-RADS score, category A-D) were extracted from the medical records.

#### 2.2.3 Genotyping

The SNP array platform used was a customized version of the Illumina BeadChip GSA MD, v3 (Illumina GSA Arrays “Infinium iSelect 24 × 1 HTS Custom BeadChip Kit” – version GOALL). This array was used to determine the 313 BC PRS SNPs, but also the *CHEK2* c.1100delC variant. Samples were processed using the manufacturer's recommended protocol. 111 of the PRS_313_ variants that could not be directly typed using this array were extracted after imputation against the 1000 genomes reference panel using Minimac [28]. PRS_313_ was standardized to the mean and SD from the population controls included in the total data set from the BCAC study [29], which were −0.424 and 0.611 respectively. PRS_311_ was calculated using a mean of −0,096 and SD of 0.609 [27]. The *CHEK2* c.1100delC variant status measured by GSA was compared to the result with the variant status that was retrieved from diagnostic DNA laboratories.

### 2.3 Statistical analysis

We tested for statistical differences (*P*-value <0.05) of characteristics between heterozygotes and non-carriers using an ANOVA test for continuous data and Chi-square tests for categorical data. Analyses were performed with STATA (version 17.0) and R (version 4.2.2).

#### 2.3.1 Cumulative risk score calculation

The LTBCR was calculated in the CanRisk tool [version 2.0]. Women were categorized in three risk groups according to the Dutch guidelines [10] by considering risk at general population level if LTBCR was under 20%, moderate risk if LTBCR was between 20% and 30%, and high risk when LTBCR was above 30%. Also, we evaluated if women would reach a LTBCR of above 50%, as this threshold is often used in unaffected heterozygote carriers to determine eligibility for preventive surgery. In the LTBCR calculations, we included risk factors stepwise, starting with FH retrieved from the pedigree and the *CHEK2* status. Risk estimation using these two variables was considered the reference, since this is currently used in clinical practice. Our main interest was to address the effect of adding information regarding the PRS_311_, QRFs and BD by considering three different approaches: (1) adding PRS_311_, as this information could be retrieved from the same blood sample and does not require any more information; (2) adding information that could be gathered directly from the individual during genetic testing and counselling, including PRS_311_ and QRFs; and (3) adding all available information, including BD, which was available in our study for 40 heterozygotes and 18 non-carriers. Reasons for missing BD information included (1) not being able to retrieve information from the surveillance hospitals indicated in the questionnaire (n = 13); (2) being younger than 35 years at time of data collection and therefore no candidate for mammographic surveillance (n = 11); (3) eligible for participating in the Dutch population-based screening program, where we could not retrieve BD information at time of data collection (n = 14); and (4) no information on whether women were under BC surveillance or the location of BC surveillance (n = 21).

## 3 Results

### 3.1 Characteristics of the study population

Table 1 shows the demographic characteristics and risk factors of heterozygotes and non-carriers. Heterozygotes and non-carriers were similar in terms of all established risk factors, except for the use of hormone replacement therapy, which was more common in non-carriers (9.6% in non-carriers and 0% in carriers; *P*-value = 0.02). Overall, over 70% of the women were a first-degree relative of the index case. Furthermore, the *CHEK2* status as determined by the GSA was always coincident with the *CHEK2* status as determined in the diagnostic setting.

**Table 1:** Characteristics of all women (heterozygotes and non-carriers of the familial *CHEK2* c.1100delC variant) included in this study.

Number		Heterozygotes	Non-carriers	
		58	59	*P*-value
Center, n (%)	AVL	22 (37.9)	26 (44.1)	0.50
	Erasmus MC	36 (62.1)	33 (55.9)	
Birth cohort, n (%)	<1960	4 (6.9)	6 (10.1)	0.69
	1960–1970	16 (27.6)	18 (30.5)	
	1970–1980	22 (37.9)	17 (28.8)	
	1980–1990	11 (19.0)	15 (25.4)	
	>1990	5 (8.6)	3 (5.1)	
Distant relative from the index in pedigree, n (%)	First-degree	42 (72.4)	42 (71.2)	0.98
	Second-degree	10 (17.2)	11 (18.6)	
	Third-degree	6 (10.3)	6 (10.2)	
Age at time of genetic testing	Mean ± SD	43.7 ± 10.2	45.9 ± 11.3	0.27
**RISK FACTORS**				
Height, cm	Mean ± SD	172.7 ± 6.0	171.0 ± 5.4	0.15
	Unknown	8	12	
Alcohol use, grams per day	None, n (%)	25 (48.1)	26 (50.0)	1.00
	Mean ± SD	83.7 ± 57.5	96.6 ± 65.5	0.48
	Unknown	9	7	
Age menarche, years	Mean ± SD	13.0 ± 1.5	12.9 ± 1.3	0.67
	Unknown	8	8	
Age menopause, years	Premenopausal, n (%)	34 (65.4)	28 (56.0)	0.33
	Mean ± SD	46.6 ± 9.0	46.3 ± 7.6	0.89
	Unknown	7	9	
Number of children, n (%)	0	8 (15.4)	9 (17.7)	0.96
	1	5 (9.6)	6 (11.8)	
	2	26 (50.0)	25 (49.0)	
	>2	13 (25.0)	11 (21.6)	
	Unknown	6	8	
Age at first childbirth, years	Mean ± SD	31.6 ± 4.4	30.6 ± 3.8	0.25
	No children	8	9	
	Unknown	8	5	
Use of oral contraception, n (%)	Never	2 (3.9)	1 (2.0)	0.57
	Ever	50 (96.2)	50 (98.0)	
	Unknown	6	8	
Use hormone replacement therapy, n (%)	Never	52 (100.0)	46 (90.4)	0.02
	Ever	–	5 (9.6)	
	Unknown	6	7	
Body mass index, kg/m^2^	Mean ± SD	25.5 ± 4.9	26.2 ± 4.0	0.45
	Unknown	9	12	
Standardized PRS_313_, Z-score	Mean	0.479	0.444	0.84
	SD	1.002	0.851	
Raw PRS_313_	Mean	−0.132	−0.153	0.84
	SD	0.612	0.520	
Standardized PRS_311_, Z-score	Mean	0.197	0.456	0.13
	SD	0.988	0.848	
Raw PRS_311_	Mean	0.028	0.186	0.13
	SD	0.601	0.516	
Breast density, n (%)	BIRADS A	2 (5.0)	1 (5.6)	0.87
	BIRADS B	15 (37.5)	8 (44.4)	
	BIRADS C	15 (37.5)	7 (38.9)	
	BIRADS D	8 (20.0)	2 (11.1)	
	Unknown	18	41	

PRS_313_ = polygenic breast cancer risk score based on 313 variants; PRS_311_ = alternative polygenic risk score based on 311 BC-associated variants. *Percentages may not add up to 100% due to rounding.

### 3.2 Stepwise addition of risk factors and BC risk distribution

**Fig. 2:**
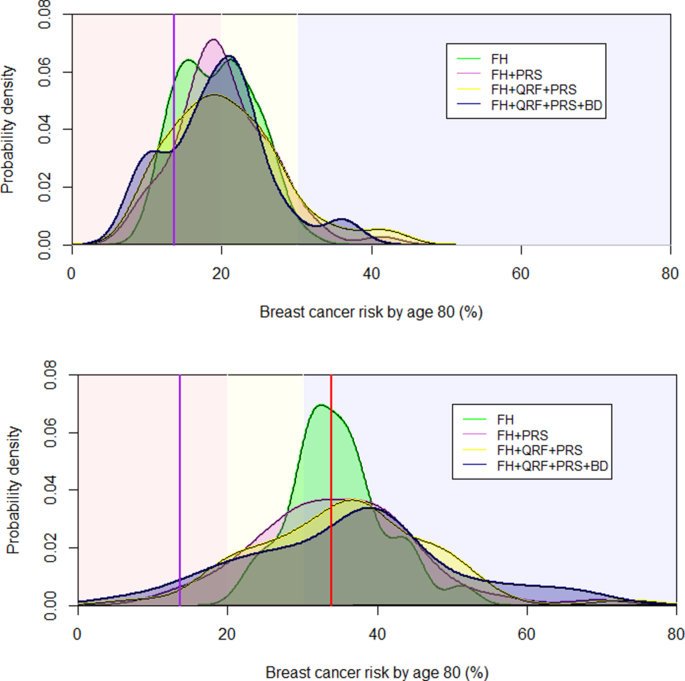
**Distribution of life-time breast cancer risk in *CHEK2* c.1100delC heterozygotes (A) and non-carriers (B) calculated using BOADICEA version 6 with stepwise inclusion of risk factors.** The background of the graphs are shaded indicating the Dutch lifetime breast cancer risk categories for women [10]: red = population risk (≤20%); yellow = moderate risk (>20% and <30%); blue = high risk (≥30%). The vertical purple line indicates the average life-time breast cancer risks for the Dutch population the vertical red line that for *CHEK2* c.1100delC heterozygotes. FH = family history; QRF = questionnaire-based risk factors; PRS = polygenic risk score based on 311 variants; BD = breast density based on BIRADS.

Fig. 2 shows the LTBCR distribution predicted in CanRisk for heterozygotes and non-carriers, separately. The curves represent the distribution of LTBCR that was calculated. The background of the graphs is shaded indicating the Dutch risk categories for women with a FH of BC [10]. The estimated LTBCR based on FH and *CHEK2* status (green line labelled: FH) ranged from 22.1% to 51.7% in heterozygotes, and from 10.7% to 31.0% in non-carriers. In addition, the distribution of the estimated LTBCR became wider meaning that we obtained better risk stratification when incorporating the PRS (pink line labelled: FH + PRS). The addition of QRFs provided an additional improvement in risk distribution (yellow line labelled: FH + QRF + PRS). Based on 40 heterozygotes and 18 non-carriers with BD information, the curves show that the variation in risk is largest when including all available information in the model (purple line labelled: FH + QRF + PRS + BD), especially in the heterozygotes.

### 3.3 Stepwise addition of risk factors and reclassification of risk category

We compared the LTBCR calculation that is mostly used in current clinical practice (including FH and *CHEK2* status) with a second calculation adding PRS_311_ and a third calculation including all information that could be gathered during genetic testing and counselling (also including PRS_311_ and QRFs) (Table 2). The majority, but not all, of the non-carriers would have been categorized at population risk level, while most of heterozygotes would have been classified as high risk. Adding PRS_311_ resulted in a reclassification of risk category for 34.5% of the heterozygotes and 35.6% of the non-carriers compared to the calculation including only FH. Of those who changed risk category, 85.0% of the heterozygotes would be recommended less intensive surveillance, while this was true for 47.6% of the non-carriers. Moreover, adding QRFs resulted in a shift in 34.5% of the heterozygotes and 45.3% of the non-carriers compared to the calculation only including FH. The addition of QRFs to the calculation based on FH and PRS_311_ contributed to a shift in 20.0% of the heterozygotes and 15.1% of the non-carriers.

**Table 2:** Comparison of risk group categorization between the life-time breast cancer risk calculation including family history and mutation status (FH), the calculation also including PRS_311_ (FH + PRS), and the calculation including questionnaire-based risk factors and PRS (FH + QRF + PRS).

**Lifetime breast cancer risk**	FH		FH + PRS		FH + PRS + QRF^†^	
	CHEK2	non-CHEK2	CHEK2	non-CHEK2	CHEK2	non-CHEK2
≤20%	0	30	5	31	6	31
20–30%	9	28	15	25	12	22
≥30%	49	1	38	3	40	6
Of whom >50%	2	0	3	0	4	0
Total number	58	59	58	59	58	59
**Shifts in life-time risk category**						
From FH to other, n (%)			20 (34.5)	21 (35.6)	19 (34.5)	24 (45.3)
From FH + PRS to other, n (%)					11 (20.0)	8 (15.1)
**Screening category change compared to FH**						
Upscaled, n (%)			3 (15.0)	11 (52.4)	4 (21.1)	14 (58.3)
Downscaled, n (%)			17 (85.0)	10 (47.6)	15 (78.9)	10 (41.7)
**Screening category change compared to FH** + **PRS**						
Upscaled, n (%)					5 (45.5)	5 (62.5)
Downscaled, n (%)					6 (55.5)	3 (27.5)

Differences in risk groups are shown for heterozygotes and non-carriers separately. The numbers of shifts might be different than expected from the presented data, as individual changes in risk category are not visible within this Table. ^†^ for three heterozygotes and six non-carriers, relevant QRFs were not available, and therefore only data on FH + PRS were used and by defintion they did not change category.

Furthermore, we compared the risk group categorization of women from whom we were able to retrieve BD information (Table 3). Adding the QRFs and BD to the calculations based only on FH and PRS_311_, resulted in a shift in 20.5% of the heterozygotes (62.5% upscaled), and 37.8% of the non-carriers (40.0% upscaled). The addition of BD alongside FH, PRS_311_ and QRFs contributed to a shift in 23.1% of the heterozygotes and 27.8% of the non-carriers.

**Table 3:** Comparison of risk group categorization between the life-time breast cancer risk calculation including family history and mutation status and PRS_311_ (FH + PRS), the calculation including questionnaire-based risk factors and PRS (FH + QRF + PRS), and the calculation also including breast density (FH + QRF + PRS + BD) in women from whom breast density information was available.

**Life-time breast cancer risk**	FH + PRS		FH + PRS + QRF^a^		FH + PRS + QRF + BD	
	CHEK2	non-CHEK2	CHEK2	non-CHEK2	CHEK2	non-CHEK2
≤20%	2	8	3	8	5	9
20–30%	11	10	9	9	8	8
≥30%	27	0	28	1	27	1
Of whom >50%	3	0	4	0	6	0
Total number	40	18	40	18	40	18
**Shifts in life-time risk category**						
From FH + PRS to other, n (%)			8 (20.5)	5 (27.8)	8 (20.5)	5 (27.8)
From FH + QRF + PRS to other, n (%)^a^					9 (23.1)	5 (27.8)
**Screening category change compared to FH** + **PRS**						
Upscaled, n (%) *			4 (50.0)	3 (60.0)	5 (62.5)	2 (40.0)
Downscaled, n (%)*			4 (50.0)	2 (40.0)	3 (37.5)	3 (60.0)
**Screening category change compared to FH** + **PRS** + **QRF**						
Upscaled, n (%) *					3 (33.3)	2 (40.0)
Downscaled, n (%)*					6 (66.6)	3 (60.0)

Differences in risk groups are shown for heterozygotes and non-carriers separately. The numbers of shifts might be different than expected from the presented data, as individual changes in risk category are not visible within this Table.

a for one heterozygotes, relevant QRFs were not available, and therefore only data on FH + PRS were used and by definition they did not change category.

Finally, we found that some heterozygotes would exceed the 50% threshold, making them eligible to discuss preventive surgery. In this study, the number of heterozygotes exceeding the 50% threshold increased after more information was included in the model (Table 2: two heterozygotes for FH alone, three when adding PRS_311_, four when also considering QRFs on top of FH and PRS_311_ and six after adding BD).

### 3.4 BC risk stratification using PRS_313_ versus PRS_311_

Comparing the model including FH and PRS_311_ with the model including FH and PRS_313_ showed that the majority of the women did not change risk category. In total, four heterozygotes (6.8%) and one non-carriers (1.7%) had an overestimation of risk category when PRS_313_ was used instead of the PRS_311_ (Supplementary Table 1).

### 3.5 Risk stratification separated by variant status and distant relatives

Based on FH alone, first-, second-, and third-degree relatives were classified in all risk categories. Adding PRS_311_ resulted in reclassifications of risk categories within first-degree relatives (in 12/42 heterozygotes and 15/42 non-carriers), second-degree relatives (in 4/10 heterozygotes and 4/11 non-carriers), and third-degree relatives (in 4/6 heterozygotes and 2/6 non-carriers) (Supplementary Table 2).

## 4 Discussion

This study showed the potential clinical value of adding known risk factors such as FH*, CHEK2* status, PRS_311_, QRFs, and when available, BD to the validated CanRisk tool in personalized BC risk prediction for unaffected heterozygotes and non-carriers of the *CHEK2* variant. By incorporating the PRS_311_ along with QRFs, a better stratification of risk and a potential improvement of clinical management was gained. We identified women who had an LTBCR exceeding 50% and would be eligible for consideration of preventive surgery, which is currently not advised in *CHEK2* heterozygotes according to international guidelines [[30], [31], [32]]. Addition of the PRS_311_ and QRFs compared to FH and *CHEK2* status alone resulted in the reclassification of risk category for more than one third of the women. The majority of reallocated heterozygotes would be downscaled (78.9%) and therefore have a later start or lower frequency of screening, whereas most of the non-carriers (58.3%) would begin surveillance earlier.

Incorporating the PRS into the CanRisk tool had the greatest impact on BC risk prediction, which is in line with previous studies [11,16,[23], [24], [25],^33^]. Here, 34.5% of the heterozygotes and 35.6% of the non-carriers would have been managed differently if the PRS_311_ had also been included in the model. These percentages are lower than found in another Dutch study, showing that 19 out of the 31 unaffected heterozygotes (58.1%) would receive different screening advice, of whom 6 (31.6%) a more intensive surveillance [26]. This might be explained by the use of the PRS_311_ versus PRS_313_, as we showed that the use of PRS_311_ alongside FH did result in lower BC risk category, especially among heterozygotes. As this is the first study conducted using the PRS_311_ in *CHEK2* families, we expect to find smaller effects than described in previous studies that used the PRS_313_.

A recent validation of BOADICEA v6 in an independent prospective population-based cohort showed that PRS contributed most to risk stratification followed by BD and finally the QRFs [33], which is in line with our results. Despite the low number of women with information on BD, we found a meaningful extra shift in about one fifth of the women when adding BD on top of the other factors. In addition, although the reported impact of adding QRFs to the model was modest [11,15], their consideration could lead to eventual significant changes in risk prediction, as many are modifiable factors over time. Therefore, special attention should be given while recommending screening for young women under the age of 35 with low PRS and high risk based solely on breast density and nulliparity, as both factors could change over time resulting in a lower BC risk. More research is needed to determine and standardize the best moment for recalculation and the clinical utility of risk-adjusted surveillance recommendations over time.

Currently, discussions in the clinical genetic setting are ongoing on to what extent relatives from *CHEK2* families should be tested, as further distant relatives would likely shift more to population-based BC risk. Although the numbers are small, this is not in line with our results. Similar risk stratifications were found in all distant relatives, also after including other risk factors. On the whole, these results do not support the choice for modified cascade screening in *CHEK2* families.

This study had some limitations. First, changes in lifestyle, reproductive and hormonal information might have occurred from the moment of completing the questionnaire until entering the data into CanRisk. Changes in the use of oral contraceptives, (number) of births, and use of hormone replacement therapy are subject to change during life. However, it is unlikely that this would have great impact on their surveillance advice, especially for women over 50 years for whom changes in surveillance are limited.

Furthermore, BD was only available for 58 women. While evidence is being gathered regarding benefits of implementing PRS in clinical practice, discussion should continue on the importance of considering all evaluable modifiable information, including BD [34,35] and QRFs when offering surveillance, especially with regard to BD due to its nature as a moderate risk factor.

From a clinical point of view, there is a need for personalized risk stratified BC surveillance instead of surveillance programs, which results in less overdiagnoses and lower costs of surveillance, without affecting the quality-adjusted life-years gained and maintaining reduced BC death [[36], [37], [38], [39]]. We showed that adding more information to the model would result in a more stratified BC risk. However, there are some barriers to overcome before implementing this in clinical practice. The addition of PRS_311_ had the most impact on stratifying BC risk. Incorporating this in clinical counselling would be wise in nearby future. Second, even though QRFs vary over time, its inclusion did impact management in a substantial 10–20% of women. To reduce the burden for genetic counsellors of (manually) entering this information into the model, a public-facing app (MyCanRisk) is currently being developed, allowing people to provide relevant information including QRFs before counselling. This will provide genetic counsellors the information needed for BC risk prediction.

Moreover, the inclusion of BD would be preferred to optimize surveillance recommendations for some women, especially young women who are more likely to have dense breast tissue [15,40]. While BD contributes to large shifts in BC risk categories, we should also keep in mind that BD will decrease over time, e.g. due to (post-)menopausal status, likely resulting in lower BC risk prediction. Therefore, age-dependent risk calculations, or 10-year risk calculations, would be preferable in women with dense breasts and a low risk based on genetic information only.

Overall, this study supports the potential clinical impact of personalized BC risk prediction using CanRisk on individual surveillance advice for women from *CHEK2* families as part of clinical genetic counselling. The BC risk stratification observed in distant relatives, may contribute to ongoing discussions on the extent of cascade genetic screening in *CHEK2* families. Finally, the pros and cons of scaling up and down surveillance programs need to be carefully weighed before implementing in clinical practice.

## Ethics approval

This research was reviewed by the Institutional Review Board at the Antoni van Leeuwenhoek Hospital - Netherlands Cancer Institute (IRBdm19-043). All data were de-identified prior to analysis.

## Funding statement

This research was supported by KWF (project 10758).

## Author contributions

All authors contributed to study conception, design, data curation and methodology. Formal analysis were conducted by Maartje A.C. Schreurs, Teresa Ramon y Cajal and Jeroen van Rooij. Funding acquisition was done by Muriel A. Adank, Antoinette Hollestelle, Marjanka K. Schmidt and Maartje J Hooning. The first draft of the manuscript was written by Maartje A.C. Schreurs and Teresa Ramon y Cajal, supervised by Muriel A. Adank, Marjanka K. Schmidt and Maartje J. Hooning. All authors commented on previous versions of the manuscript, read and approved the final manuscript.

## Data availability

The data sets generated and/or analyzed during this study are available to non-commercial parties upon reasonable request from the corresponding author. Only anonymized data will be transferred.

## Consent to participate

Informed consent was obtained from all individual participants included in the study.

## Declaration of competing interest

Authors declare no potential conflict of interest.
