# Supplementary material for: The benefit of adding polygenic risk scores, lifestyle factors, and breast density to family history and genetic status for breast cancer risk and surveillance classification of unaffected women from germline CHEK2 c.1100delC families
Source: Breast. 2024 Apr 12;75:103724. doi: 10.1016/j.breast.2024.103724 (PMC11127622; doi:10.1016/j.breast.2024.103724)
Supplement: Multimedia component 1 [file mmc1.docx]

**Supplemetary Table 1: Comparison of risk group categorization between the life-time breast cancer risk calculations based on family history and PRS_313_ vs family history and PRS_311_.**

| **Life-time breast cancer risk** | | FH + PRS_311_ | | | | | |  | |
| --- | --- | --- | --- | --- | --- | --- | --- | --- | --- |
|  |  | **≤20%** | | **20-30%** | | **≥30%** | | Total | |
|  |  | CHEK2 | non-CHEK2 | CHEK2 | non-CHEK2 | CHEK2 | non-CHEK2 | CHEK2 | non-CHEK2 |
| FH + PRS_313_ | **≤20%** | 4 | 30 | 0 | 0 | 0 | 0 | 4 | 30 |
|  | **20-30%** | 1 | 1 | 12 | 25 | 0 | 0 | 13 | 26 |
|  | **≥30%** | 0 | 0 | 3 | 0 | 38 | 3 | 41 | 3 |
|  | Total | 5 | 31 | 15 | 25 | 38 | 3 |  | |

| Life-time breast cancer risk | | FH | | FH+PRS | | FH +PRS+QRFs^†^ | |
| --- | --- | --- | --- | --- | --- | --- | --- |
|  |  | CHEK2 | non-CHEK2 | CHEK2 | non-CHEK2 | CHEK2 | non-CHEK2 |
| ≤20% | FDR | 0 | 20 | 3 | 20 | 3 | 21 |
|  | SDR | 0 | 7 | 1 | 7 | 1 | 6 |
|  | TDR | 0 | 3 | 1 | 4 | 2 | 4 |
| 20-30% | FDR | 3 | 21 | 9 | 20 | 6 | 17 |
|  | SDR | 3 | 4 | 3 | 4 | 5 | 4 |
|  | TDR | 3 | 3 | 3 | 1 | 1 | 1 |
| ≥30% | FDR | 39 | 1 | 30 | 2 | 33 | 4 |
|  | SDR | 7 | 0 | 6 | 0 | 4 | 1 |
|  | TDR | 3 | 0 | 2 | 1 | 3 | 1 |
| Of whom >50% | | 2 | 0 | 5 | 0 | 12 | 0 |
| Total, N | | 58 | 59 | 58 | 59 | 58 | 59 |
| shift compared to FH, n(%)  FDR  SDR  TDR | |  |  | 12 (28.6)  4 (40.0)  4 (66.6) | 15 (35.7)  4 (36.4)  2 (33.3) | 9 (23.1)  6 (60.0)  4 (66.6) | 16 (42.1)  6 (60.0)  2 (40.0) |

**Supplementary Table 2: Comparison of risk group categorization between the LTBCR calculation including family history and mutation status (FH), the calculation also including PRS_311_ (FH+PRS), and the calculation including questionnaire-based risk factors and PRS (FH+QRF+PRS) for first-degree relatives (FDR, n=42 heterozygotes and n=42 non-carriers), second-degree relatives (SDR, n=10 heterozygotes and n=11 non-carriers) and third-degree relatives of the index (TDR, n=6 heterozygotes and n=6 non-carriers).**

Differences in risk groups are shown for heterozygotes and non-carriers separately.  ^†^ for three heterozygote FDR, four non-carrier FDR, one non-carrier SDR, and one non-carrier TDR, relevant QRFs were not available, and therefore only data on FH+PRS were used and by definition they did not change category.
